# BOOSTING BRAIN WAVES IMPROVES MEMORY

**DOI:** 10.3389/frym.2021.605677

**Published:** 2021-11-22

**Authors:** Richard J. Addante, Mairy Yousif, Rosemarie Valencia, Constance Greenwood, Raechel Marino

**Affiliations:** 1 Department of Psychology, Florida Institute of Technology, Melbourne, FL, United States; 2 Department of Psychology, California State University, San Bernardino, San Bernardino, CA, United States

## Abstract

Have you ever wanted to improve your memory? Or have you struggled to remember what you studied? Memory uses special patterns of activity in the brain. This experiment tested a new way to create brain wave patterns that help with memory. We wanted to see if we could improve memory by using lights and sounds that teach the brain waves to be in sync. People wore special goggles that made flashes of light and headphones that made beeping noises. This trained the brain through a process called entrainment. The entrainment put the brain in sync at a specific brain wave pattern called theta. People whose brains were trained to be in theta had better memory compared to people whose brains did not get trained. We learned that entrainment is a cool new way to make memory better.

## Graphical Abstract

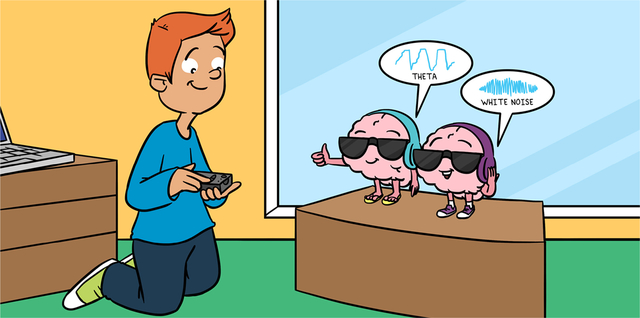

## MEMORY AND BRAIN WAVES

Remembering is important for everyday life. It helps us with lots of things, such as remembering to do homework or knowing the answers for a test. Most times forgetting is normal, but occasionally it can be caused by damage to the brain or certain diseases. Our experiments studied the long-term version of memory, which includes things like remembering your first day of school or a friend’s birthday party. These memories happen in the brain due to the activity patterns of brain cells in different brain areas, working together to remember. The brain cells, called **neurons**, send electrical signals back and forth in just the right sequences, like friends throwing a ball back and forth. The brain is made of millions of neurons, and their patterns of electrical activity are called brainwaves. Brain waves are like waves at the beach: they can be big or small, and fast or slow. The speed of the brain waves is called **frequency**, measured in a unit called **hertz** (Hz). Hertz tells us how many times the brain waves happen in 1 s. But to make it easier, brain waves are usually referred to by names. One of the slowest brainwaves is called **theta** and it occurs four to eight times per second (4–8 Hz). Theta happens while people are very relaxed or during sleep. A faster brainwave, called **beta**, occurs twelve to sixteen times per second (12–16 Hz). Beta is usually found when people are awake and conscious.

**NEURON**

Cells within the nervous system, including cells in the brain and the spinal cord, that send (and receive) electrical messages with other cells.

**FREQUENCY**

In brain recording, how many times a signal (or wave) happens in a second.

**HERTZ**

The basic unit of measure for frequency, or how many times a signal (or wave) happens in a second. It is abbreviated as (Hz).

**THETA BRAIN WAVES**

Slow brainwaves, with a frequency of 4–6 Hz, which are common in sleep, relaxation, and deep meditation.

**BETA BRAIN WAVES**

Fast brainwaves, with a frequency of 12–16 Hz, which are commonly associated with being awake and conscious.

## CAN THE BRAIN BE TRAINED TO HAVE CERTAIN BRAIN WAVES?

The rhythmic patterns of brain activity can be changed through a process known as **entrainment**. That is, we can train the brain to work at different frequencies. This is like getting a musical band to play together at a faster or slower speed, or like tuning into a specific radio station. Entrainment works by giving the brain inputs, like sounds and lights, at the frequency we want it to be in. We can do this using goggles that flicker lights at the exact frequencies that we want the brain to be in, or we can use headphones to play sounds (beeps) at just the right frequency. These methods can entrain the brain without hurting it.

**ENTRAINMENT**

Synchronization of brainwaves with external stimuli, which can include pulses of light and sound at specific frequencies. Entrainment guides the brain gently and safely into specific brain wave patterns.

In our study, we wanted to see if entrainment could improve memory by boosting the theta waves. We got the idea to do this because our prior studies had shown that theta waves happen just *before* a person remembers something correctly [1]. This was surprising and exciting to scientists, because our finding meant it could be possible to improve a person’s memory by changing the brain activity happening right before remembering—imagine that! Could boosting theta waves actually improve what people remember? We predicted that boosting theta waves by audio-visual entrainment would result in improved memory.

## HOW DID WE TEST OUR PREDICTIONS?

**Figure 1:**
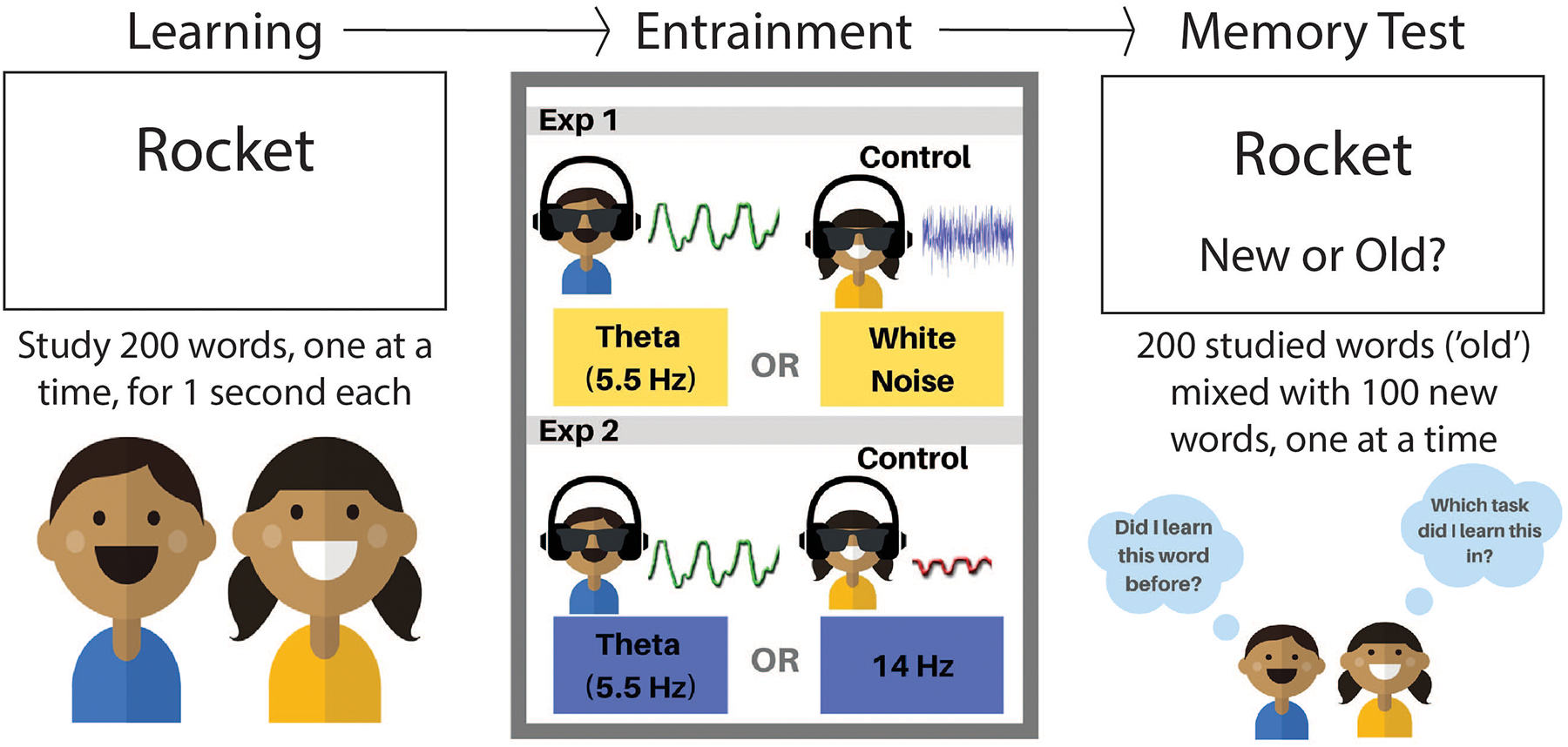
There were three parts of our memory experiment. First, people learned words on a computer screen, one at a time. Second, they took a break, and received 36 min of brain entrainment, consisting of lights and sounds. Third, they took a memory test, in which they saw a bunch of the words they studied, mixed in with new words. They were asked to remember whether they had seen each word before, classifying each as “old” or “new.”

Two experiments were done to see if boosting theta waves would improve memory. The first experiment compared a boost of theta waves to a boost of random patterns, called **white noise**. The second compared boosting theta waves to boosting beta waves. In both experiments, people first studied a list of 200 words, one at a time. Later they had a memory test in which those same words were mixed with 100 new words. As words appeared before them one at a time, the participants were asked to remember whether each word was had been studied in the first part of the experiment. In between the studying and the memory test, there was a 36-min period of audio-visual entrainment, using headphones, and googles as described above (Figure 1). The goggles blocked out everything except the flickering lights and the headphones blocked all sounds except those being presented. We could control the volume of the sounds and the brightness of lights. The type of audio-visual stimulation depended on the group people were assigned to—participants saw and heard either theta, beta, or random patterns.

**WHITE NOISE**

A random signal like the “snowy” view on a television screen with no signal or a scratchy radio station on which you hear only static sounds.

In the first experiment, 50 people (26 females, 24 males, aged 18–25) were tested. They were divided equally into two groups. The experimental group received lights and sounds that boosted theta waves (5 Hz). The control group received a random pattern of flickering lights and sounds (white noise), and this group was used as a comparison. None of the participants knew which group they were placed in. In the second experiment, 40 new people were tested (22 females, 18 males, aged 18–26). Again, they were separated into two groups that received different entrainment patterns. This time, one group received theta entrainment (5 Hz) and the other group received beta entrainment (14 Hz). This was done to see if training other brain patterns would also improve memory, or if memory improvement was specific to theta.

But how can we visualize what is going on in the participants’ brains without getting inside their heads? **Electroencephalography (EEG)** is a safe way to measure the electrical activity produced by brain waves, without surgery. It simply involves placing a cap with sensors called electrodes on a person’s head “Measuring Brain Waves in the Classroom” [2]. Scientists can compare EEG results when people remember something successfully vs. when they forget. The difference in the EEG readout tells us what the brain activity for memory looks like. Our earlier studies found that theta waves in the front part of the brain predicted successful memory. So, in these experiments, we also looked for theta activity in the front of the brain during the time of the experiments when people were remembering.

**ELECTROENCEPHAL-OGRAM (EEG)**

A technique for measuring the electrical activity of the brain, using a cap with sensors called electrodes, which is worn over a person’s head.

## WAS OUR PREDICTION CORRECT? (SPOILER ALERT: YES!)

**Figure 2:**
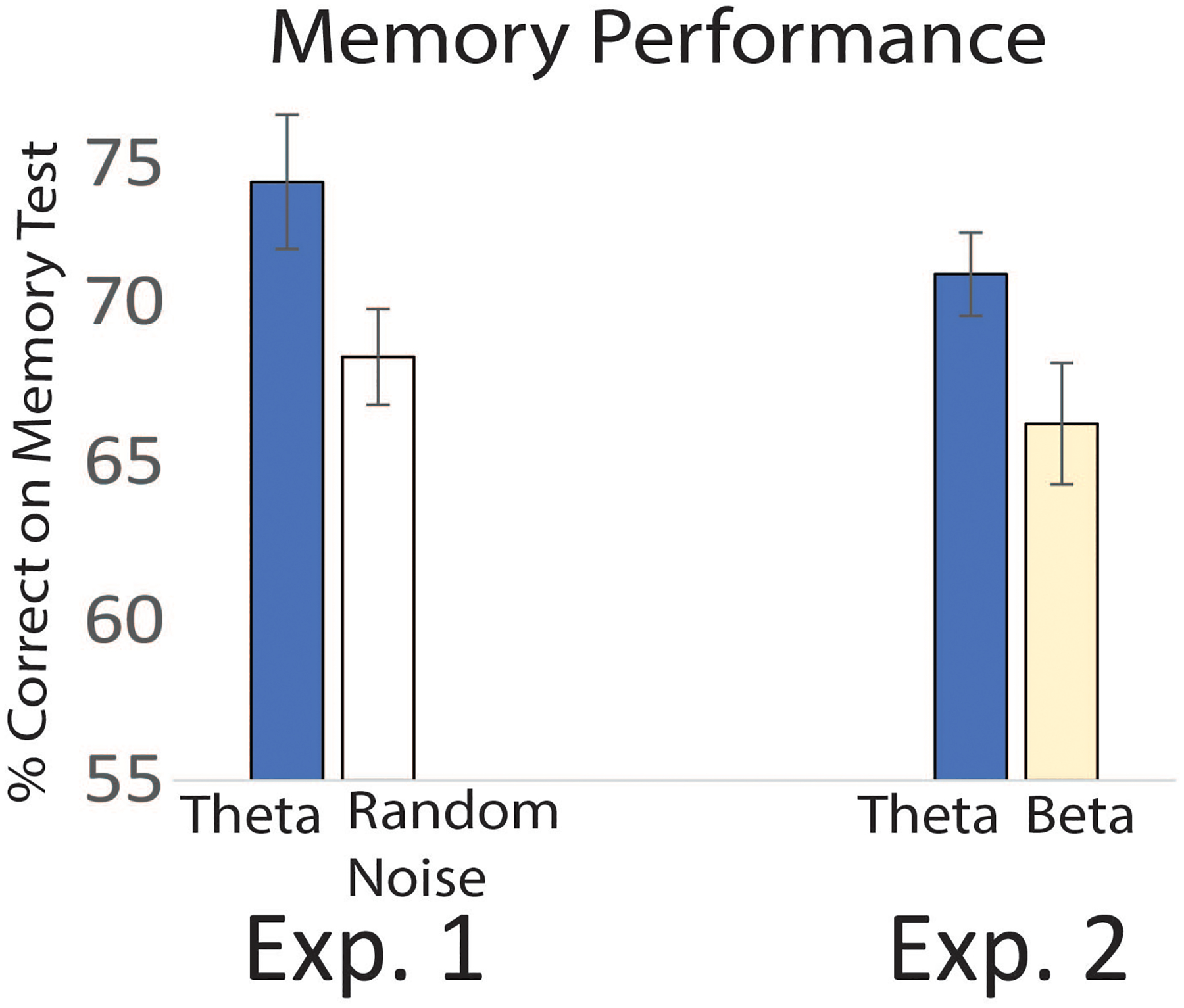
In the first experiment, the group that received theta brain entrainment scored better on the memory test compared to the group that received white noise. In the second experiment, the group that received theta entrainment showed better memory results than the group that received beta entrainment. The difference between entrainment conditions was statistically significant, which means that these differences had a <5% probability of being due to random chance. The lines on the top of each bar represent the standard error of the mean, and they show how much the data points vary from the average.

Our first experiment showed that the group receiving theta entrainment had better memory than the group receiving random noise (Figure 2). This suggested that audio-visual stimulation in the theta range improved memory. But how do we know that it was theta entrainment that made the difference? That is where our second experiment came in. We wanted to see if memory improved when beta waves were boosted, or whether the effect was specific for the theta group. Results of this second experiment showed that only the group receiving the theta boosts showed improvements in memory: the group receiving beta stimulation showed no memory improvement (Figure 2).

**Figure 3:**
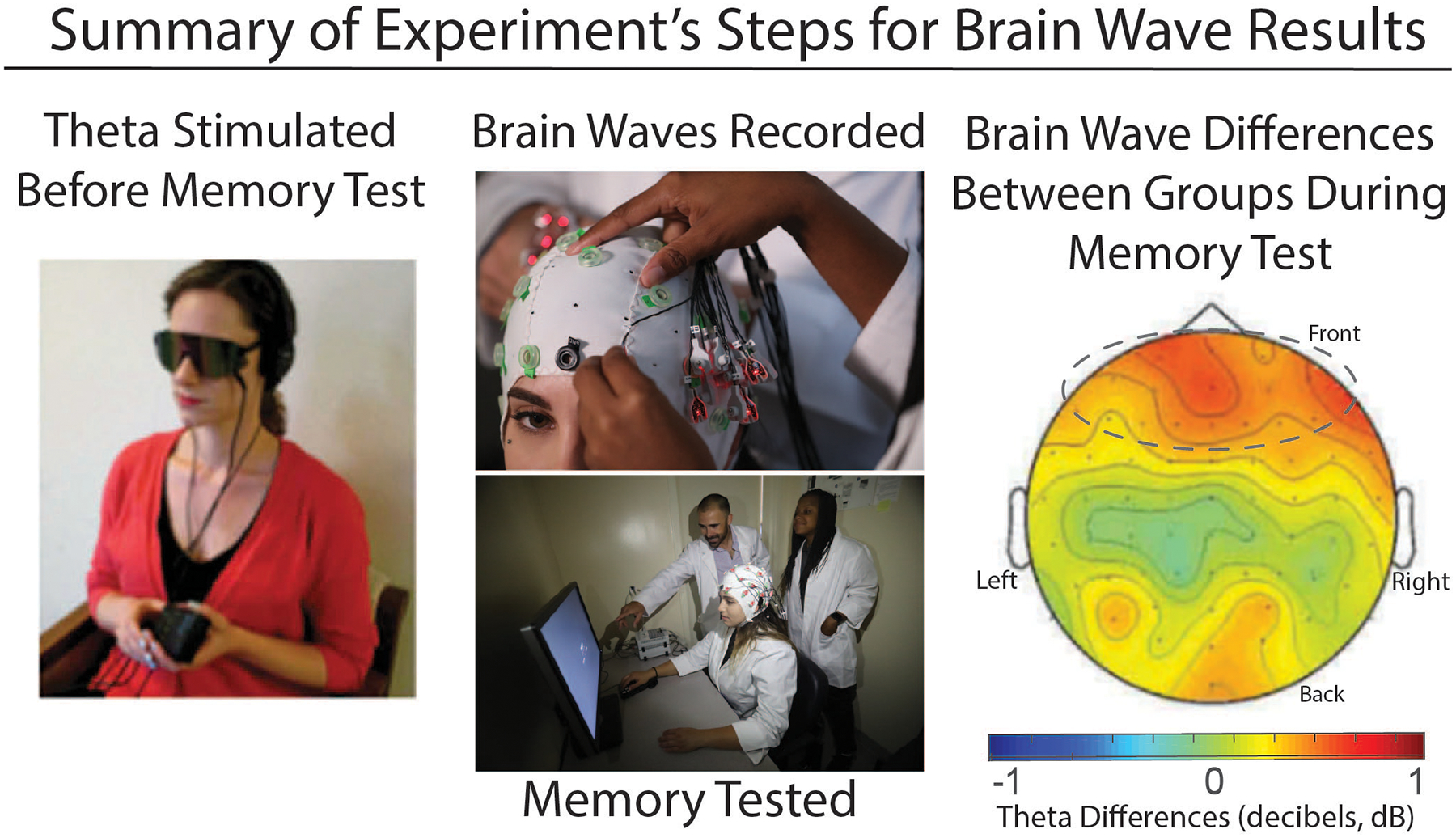
**(A)** After learning the words, participants received audio-visual entrainment of their brain waves using either theta (4–6 Hz) or beta (14 Hz) stimuli. **(B)** During the memory test, participants had their brainwaves recorded with EEG. **(C)** The group that received theta stimulation showed higher theta activity during the memory test than the beta group did. This brain map (as if we were looking down on the brain from above) theta differences during memory: subtracting between groups that received either theta or beta stimulation beforehand. The color scale shows theta activity during the memory test. The main differences were seen in the frontal sites, which are circled.

Results of the EEG showed that people’s brainwaves were boosted in the same patterns that they were receiving from the entrainment devices (Figure 3). This means that people who received theta entrainment showed increased theta waves during the memory test, and beta entrainment likewise boosted beta waves (but importantly-did not improve memory). This was important because it meant that entrainment was actually working to change the brain.

## SO WHAT?

We found that entrainment can safely manipulate brainwaves to improve memory. Every study has some strengths and weaknesses. One of the strengths of this study is that it used two experiments and good control groups for comparisons that ruled out alternative possibilities. While this is exciting, there is still much work to be done. For instance, one limitation is that we do not know the minimum amount of time needed for entrainment to work, since we only tested 36 min of entrainment. We also do not yet know how long the memory improvements last, and it is important to note that it has not been tested in kids yet and the flashing lights may not be safe for people who have epilepsy. We must also remember that although we showed that training of theta waves boosted later memory scores and also boosted theta activity in the brain during memory, this does not necessarily mean that the boosted theta activity during the memory portion of the experiment *caused* the improved memory—just that the entrainment improved both memory and later theta waves. However, other studies have shown that memory scores can be caused by different brain waves such as theta [1, 3–5].

There are many exciting potential applications of these findings in the future. One hope is that we can use entrainment devices to help people who suffer from memory disorders. For example, entrainment could possibly help older people who forget many things, people with head injuries, or those with diseases that affect memory [3–5]. Entrainment of brainwaves might also help people to improve memory performance in normal situations, too. It could help people improve in school, in sports, or in their jobs. For instance, entrainment could be used to help astronauts flying in space to avoid memory mistakes! We are currently doing research to explore these possibilities, so stay tuned—better learning and memory may be as simple as getting into the right state of mind. So, “Think Theta!”
